# Change of Muscle Architecture following Body Weight Support Treadmill Training for Persons after Subacute Stroke: Evidence from Ultrasonography

**DOI:** 10.1155/2014/270676

**Published:** 2014-03-24

**Authors:** Peng Liu, Yanjun Wang, Huijing Hu, Yurong Mao, Dongfeng Huang, Le Li

**Affiliations:** Department of Rehabilitation Medicine, The First Affiliated Hospital of Sun Yat-sen University, Guangzhou 510080, China

## Abstract

Although the body weight support treadmill training (BWSTT) in rehabilitation therapy has been appreciated for a long time, the biomechanical effects of this training on muscular system remain unclear. Ultrasonography has been suggested to be a feasible method to measure muscle morphological changes after neurological diseases such as stroke, which may help to enhance the understanding of the mechanism underlying the impaired motor function. This study investigated the muscle architectural changes of tibialis anterior and medial gastrocnemius in patients after subacute stroke by ultrasound. As expected, we found the effect of BWSTT on the muscular system. Specifically, the results showed larger pennation angle and muscle thickness of tibialis anterior and longer fascicle length of medial gastrocnemius after the training. The findings of this study suggest that the early rehabilitation training of BWSTT in subacute stage of stroke provides positive changes of the muscle architecture, leading to the potential improvement of the force generation of the muscle. This may not only help us understand changes of subacute stroke in muscular system but also have clinical implications in the evaluation of rehabilitation training after neurological insults.

## 1. Introduction

Stroke survivors often develop spasticity, contractures, muscle weakness, and decreased range of motion, which severely affect their activities of daily living [1, 2]. Three months after the onset of stroke, approximately 25% of the surviving patients are still using wheelchair, and, in 50% of the survivors, the gait velocity and endurance are considerably reduced [3]. Therefore, restoration and improvement of gait after stroke are major aspects of neurorehabilitation.

Body weight supported treadmill training (BWSTT) is a type of step training with task-specific nature and partial body weight of the subjects is held [4]. This interactive locomotor training first came from animal experiment which demonstrated recovery of locomotion and the spinalized cats could regain normal gait pattern after 1 to 3 months partial weight supported walking on treadmill [5]. Clinically, BWSTT is proved to be a promising technique for the restoration of gait in stroke and paralytic subjects [6–8]. It enables the harness-secured patients to practice numerous steps assisted by therapists at an early stage after neurological insult [6]. Previous studies have showed that BWSTT is more effective for the restoration of gait and improving walking capacity by establishing symmetric and efficient gait as compared to regular physiotherapy in people after stroke [7, 8]. However, others reported conflicted findings that BWSTT is not superior to the conventional gait training [9]. This discrepancy might relate to the limitation of evaluation methods and the limited understanding of the recovery mechanism of treadmill training.

To evaluate the effects of BWSTT, clinical tests and scales are often used [10]. Clinical scales are relatively subjective in the evaluation of the efficiency of different stroke rehabilitation programs. In addition, these evaluations do not reveal the underlying mechanisms of those interventions to the neuromuscular system. The motor recovery of limb function is related to spinal locomotor pools, which include a central pattern generator for activity of automatic, alternating flexor, and extensor lower limb muscles. Spinal locomotor pools are highly responsive to phasic segmental sensory inputs and show evidence of learning during step training [11]. Previous results showed that BWSTT could reduce the level of loading on the lower limbs and enable the human lumbosacral spinal cord to modulate efferent output in a manner that may facilitate the generation of stepping [12]. However, to our knowledge, there is still a lack of study or evidence to investigate the biomechanical mechanism of motor function improvement after BWSTT in the peripheral neuromuscular system, especially from the muscle fascicle level.

Muscle architecture, defined here as a geometrical arrangement of fascicle, affects the muscle function [13]. In a pinnate muscle, fascicles (bundles of fibres) are arranged parallel and obliquely with respect to the tendon; then the forces exerted by muscle fibers are in turn modified by this pennation angle when they are transmitted to tendon [14]. Muscle architecture therefore characterizes and specifies the force-generating capability of a muscle. Although MRI has been widely accepted as a gold standard in measuring the muscle parameters, especially cross-section area [15], it is costly and limited in cooperation with other instruments and in different testing conditions with muscle contraction [16]. As a noninvasive medical imaging technology, ultrasonography has been applied to measure human skeletal muscle architecture in vivo [17]. It is a feasible method to measure pennation angle, muscle fascicle length, and muscle thickness. Based on the normal subjects and highly-trained bodybuilders, Kawakami et al. found a significant correlation between muscle thickness and pennation angles at triceps brachii [18]. Recently, ultrasonic studies have been conducted to examine hypertonic muscles in patients with neurologic disorders [19, 20]. Our previous study showed that persons after chronic stroke had shorter muscle fascicle length at brachialis compared to unaffected side [19], and Gao and his colleagues also found smaller pennation angle and shorter muscle fibre length in gastrocnemius of chronic stroke survivors compared to age-matched healthy control [20]. However, how the muscle morphology change on persons in subacute stroke remains unclear, and how the early stage of exercise training improves muscle function needs investigation.

The purpose of this study was, therefore, to measure the muscle architectural parameters of tibialis anterior and medial gastrocnemius in patients after subacute stroke by ultrasound and to investigate their changes after BWSTT together with other clinical scores, muscle strength, and walking speed to assess the effectiveness of the intervention, which would help us understand the biomechanical mechanism of the training. We hypothesize that ultrasound could differentiate the changes after stroke and that the training effects on motor recovery after BWST might be related to the changes of muscle architecture.

## 2. Methods

### 2.1. Participants

Fifteen adults with subacute stroke (9 men, 6 women; mean, 60.5 y; age range, 51–73 y) and eight age-matched healthy subjects (5 men, 3 women; mean, 57.0 y; age range 41–75 y) were recruited in this study. The inclusion criteria for the hemiparetic subjects included (1) having hemiparesis for no more than 3 months resulting from first stroke insult; (2) presence of clinically detectable spasticity in the ankle dorsiflexor, with a Modified Ashworth Score (MAS) larger than 1 (maximal value, 4); (3) a passive range of motion in the ankle joint on the paretic side from −15° (dorsiflexed direction) to 45° (plantarflexed direction), here, 0° was defined as ankle in neutral position (the sole of the foot perpendicular to the tibia); (4) adequate mental capacity to attempt the tasks as instructed; and (5) an absence of other significant medical complications.  Table 1 shows the baseline demographic and clinical characteristics for the people after subacute stroke. This study was approved by the Human Subjects Ethics Committee of The First Affiliated Hospital of Sun Yat-sen University. All the participants gave informed consent following the ethical procedures.

### 2.2. Ultrasound Measures of Muscle Parameters

The stroke survivors were randomly assigned to conventional rehabilitative treatment plus BWSTT (BWSTT group, *n* = 8) and conventional treatment plus over-ground gait training only (CGT group, *n* = 7). A B-mode ultrasonography scanner (DP6600, Mindray Inc, China) with a 7.5 MHz, 38 mm probe (imaging resolution, 0.3 mm; frame rate, 25/s), and a hand-held dynamometer (MicroFET3, Hoggan Inc, UT, USA; with the precision of 0.4 N and range from 13 N~1330 N) were used in the present study.

During the experiment, the subjects were laid supine on a checking bed and were supported with a towel roll under ankle while hip and knee joints were in full extension [18]. During the testing for tibialis anterior (TA), the ultrasound probe was put perpendicularly to the dermal surface of central region of TA muscle, which is half-distance between the malleoli and the proximal end of the tibia, over the midsagittal plane [21]. For median gastrocnemius (MG) muscle, the probe was placed on a site on the muscle 30% proximal between the medial malleolus of the fibula and the medial condyle of the tibia [22]. The size of the probe is 38 mm and the probe was put on the muscle belly. The position of muscle belly was confirmed based on the contraction of the muscle as well as the experience of the experienced physical therapist. A marker pen was used to set the position on the skin to localize the probe position. Coupling gel was applied to enhance ultrasound conduction between the ultrasound probe and skin surface. Accuracy of the ultrasound method in measuring muscle architectural features has been previously demonstrated to show good agreement with direct anatomical measurement on cadaver [23]. The experiment consisted of two different conditions: muscle at rest and at maximum voluntary contraction (MVC). For each condition, ankle joint was measured ranging from dorsiflexion 15° to plantar flexion 45° with increments of 15° using the hand-held dynamometer and, for each position, three trails of muscle contractions were tested. We followed the similar procedure of our previous study using ultrasound measurement on muscle architecture at rest and MVC [19]. The hand-held dynamometer was held by an experienced physical therapist and the testing position is referenced with text book [24]. In the rest condition, subjects were required to relax during the measurement. In the MVC condition, subjects were instructed to take 1 or 2 s to come to maximal effort and hold for 3–5 s then the muscle strength was measured by the tester using the hand-held dynamometer. Ultrasound images were collected simultaneously. All subjects were instructed to avoid eversion-inversion and adduction-abduction of the foot during MVC. The test was performed three times with a 30 s interval to avoid muscle fatigue. Ultrasound measurements were conducted at the first of enrollment and last day after the 3-week training.

**Figure 1:**
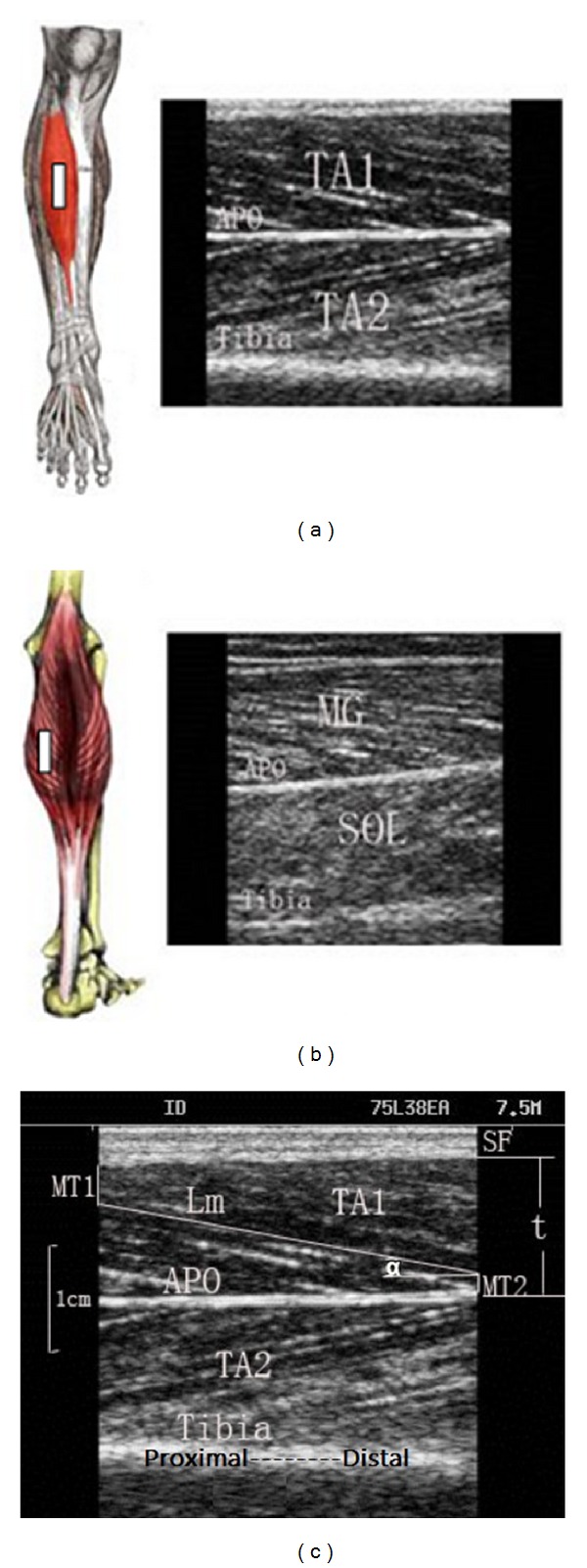
Probe positions on the measured muscles and typical ultrasound images for measurement on (a) TA and (b) MG. (c) Demonstration of the labels for muscle parameters. The bright fringe in the lower region of the image shows the muscle-tibia boundary. Aponeurosis (APO) is the boundary between the superficial and deep layer of TA. SF is subcutaneous fat. *L*_*m*_ is the visualized part of the entire muscle fascicle length and can be measured directly; MT_1_ and MT_2_ are the distance of the fiber proximal end point to the superficial aponeurosis and the distance of the fiber distal end to the bone, respectively; *α* is the pennation angle; TA1 is the superficial layer of the TA; and TA2 is the deep layer of TA.

Probe position and typical ultrasound images of TA and MG are shown in Figure 1. The white fringe of the tibia bone and the dark muscle fascicle are displayed in the ultrasound image. Pennation angle (*α*) was directly measured from the image, and the entire muscle fascicle length (*L*_*f*_) was estimated using a trigonometry method by assuming a linear continuation of the muscle fascicle [19]. Consider

(1) $$\begin{array}{c} L_{f}=L_{m}+\frac{\text{M}{\text{T}}_{1}}{\sin\alpha }+\frac{\text{M}{\text{T}}_{2}}{\sin\alpha }, \end{array}$$

where *L*_*f*_ is the entire estimated muscle fascicle length, *L*_*m*_ is the visible part of the muscle fiber, and *α* is the pennation angle. MT_1_ and MT_2_ denote the distance of the fiber distal end point to the superficial aponeurosis and the distance of the fiber proximal end to the bone, which is also used to calculate muscle thickness.

### 2.3. Training Protocol

All the stroke survivors were treated in 60-minute walking training sessions every weekday for 3 weeks with a total of 15 sessions. For BWSTT, the initial BWS (body weight support) amount was set at 30%~40%, and the speed of the treadmill was set at 0.5 mph (miles per hour). We followed the training schedule both with the recommendation from literature on BWSTT [4] as well as our patients' conditions and response being evaluated by experienced physical therapist and physicians. In Hesse's study [4], he recommended that the initial body weight support should be no more than 30% BW and, during therapy, treadmill speed should be increased and body weight support reduced as soon as possible. The training intensity at the first week was around 20 mins and increased to 40 mins in the third week, while the treadmill speed increased to around 2.0 mph. For the control group, they received over-ground walking training of 60 mins daily.

For all the recruited subjects, another two-hour therapy program of nongait activity such as bed mobility, transfers, strengthening, and balance training was also scheduled as normal training at in-patient section of hospital. All physical therapists involved in the study were trained according to the protocol and documented participants' daily compliance with the protocol. In addition, the entire rehabilitation team was educated concerning the experimental study protocol to ensure compliance when participants were not working with therapy staff.

## 3. Other Outcome Measures

Besides the ultrasound measurements, other outcome measures were the muscle strength, 10 meters walking speeds, Modified Ashworth Scale (MAS), and the lower limb subscale of Fugl-Meyer assessment (FMA-LE). Assessments were made at baseline and after the treatment by an examiner who was blinded to the group information of the subjects.

### 3.1. Statistical Analysis

In this study, values for muscle architectural parameters and muscle strength were presented as mean ± SD. SPSS (version 15.0, SPSS Inc, Chicago, IL, USA) was used to compare the difference of outcome measurements. Analysis of variance (ANOVA) with Bonferroni post hoc test was used to evaluate the changes of the parameters across conditions. Independent *t* test was used to compare the data of rest and MVC condition at each specific joint angle. A paired *t* test was used to compare muscle architectural parameters, muscle strength, FMA-LE scores, and walking speed before and after the training. Rank-sum test was used to compare MAS scores before and after the training. Pearson correlation analysis was conducted between muscle architecture parameters and muscle strength. The significant level was set as 0.05 for all statistical tests.

## 4. Results

**Figure 2:**
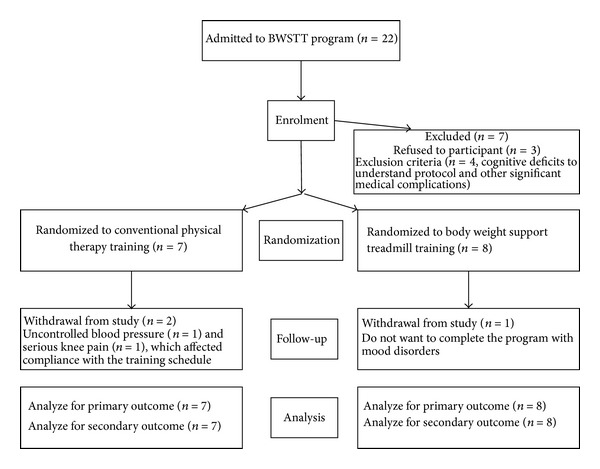
The study flow chart.

The baseline measures of common demographic variables, the lower limb subscale of Fugl-Meyer assessment, and the Modified Ashworth Score did not significantly differ between the BWST group and control group (Table 1). Subject disposition is detailed in the flow chart (Figure 2).

**Figure 3:**
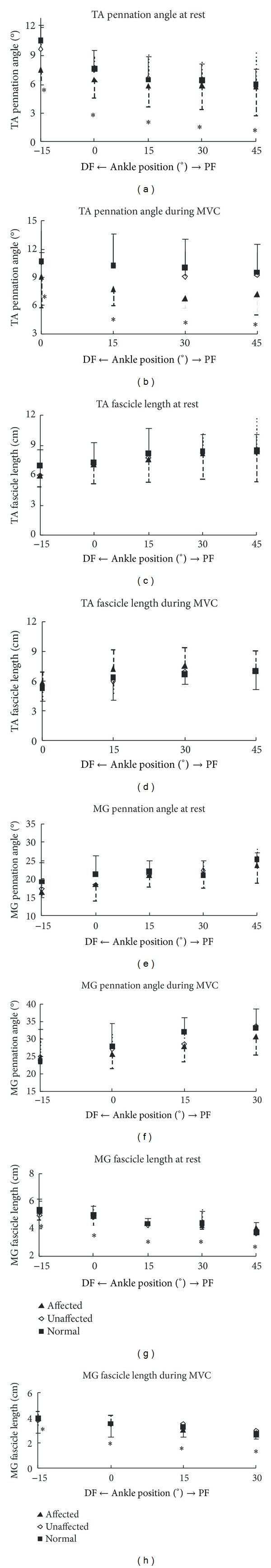
Pennation angle and fascicle length of TA ((a)–(d)) and MG ((e)–(h)) in the affected side, the unaffected side of the subjects after stroke, and the right side of healthy subjects as a function of ankle joint angle at the rest condition and MVC. The error bar represents 1 standard deviation (SD). *Any significant difference between the affected group and the unaffected group (*t* test, *P* < 0.05).

For TA muscle, it was found that the measured muscle pennation angle and fascicle length were joint-angle-dependent in all three groups at the rest and MVC condition (Figures 3(a)–3(d)). Further comparisons between groups found that the pennation angles and muscle thickness of the affected side were significantly smaller (*P* < 0.05) than the unaffected side and those of healthy group at both two conditions, whereas there was no significant difference of muscle fascicle length among the groups in rest condition. There were no significant difference of muscle thickness (*P* > 0.05) between rest and MVC (Table 2). Compared to the baseline value, pennation angle (6.15 ± 1.28°) and muscle thickness (1.02 ± 0.09 cm) at the affected side of BWSTT group at rest condition significantly increased to 7.26 ± 1.62° (*P* < 0.05) and 1.09 ± 1.12 cm (*P* < 0.05) after the training, while there were no significant differences in CGT group (Table 2). A similar trend was also shown in MVC condition. Muscle strength of dorsiflexion at affected side in BWSTT group significantly increased from 49.04 ± 28.12 N to 83.75 ± 42.72 N after the training, while there were no significant changes in the unaffected side and both two sides in CGT group (Table 3).

For the MG muscle, pennation angle and fascicle length were joint-angle-dependent in all three groups at the rest and MVC condition (Figures 3(e)–3(h)). Comparison between groups showed that the affected fascicle lengths were significantly shorter (*P* < 0.05) than the unaffected side and the healthy group. There were no significant difference of muscle thickness (*P* > 0.05) between rest and MVC (Table 2). After the training, the fascicle length of affected side (5.23 ± 1.07 cm) was significantly longer than that of baseline value (4.66 ± 1.06 cm, *P* < 0.05) (Table 2). In addition, the plantarflexors strength of affected side significantly increased from 93.67 ± 40.94 N to 115.39 ± 65.37 N after the training. However, this trend of muscle fascicle and strength was not found in unaffected side and CGT group (*P* > 0.05).

The clinical scores of affected side showed the improvement after the training. FMA-LE was significantly increased (*P* < 0.05) and MAS was significantly decreased (*P* < 0.05), while there was no significant difference in the CGT group (Table 4). 10-meter walking test of self-selected speed showed that the BWST has a significant increase (*P* < 0.05), while there were no such changes in CGT group (Table 4).

## 5. Discussion

In this study, ultrasound measurements were conducted on the TA and MG of subacute stroke survivors together with muscle strength and clinical scores before and after 3 weeks BWSTT. The results demonstrated the muscular morphological changes in larger pennation angle and muscle thickness of tibialis anterior and longer fascicle length of medial gastrocnemius after the training, as well as muscle strength. This suggests that the early rehabilitation training of BWSTT is helpful to the changes of the muscle architecture which contributes to the potential of the force generation of the muscle [25].

Our findings showed that the muscular architectural parameters in the affected side and in the unaffected side were different and were joint-angle-dependent at the rest condition. Previous study found significant decrease in pennation angle and fiber length of gastrocnemius medialis muscle at the affected side of chronic stroke survivors [20]. The immobilization of the flexor in a shortened position and increased muscle stiffness might cause these muscle architectural changes. The reason of the shorted muscle fascicle length may be due to reduction in the number of sarcomeres in the spastic muscle fiber [26] and decrease of the pennation angle related to muscle disuse [27]. During muscle maximum voluntary contraction, force generated by muscle elongates the tendon and aponeurosis, changing the architecture of muscle; that is, pennation angle increased and fascicle length shortened. This phenomenon is widely known from previous studies [15, 16, 18]. The findings in our study share agreement of the phenomenon, while fascicle length of both TA and MG has shortened and pennation angle of them has increased at MVC condition. Compared to unaffected side, smaller pennation angle and fascicle length changes were found in the affected side during isometric contraction, and these smaller changes might be due to weakness in the muscle after the onset of stroke. We found that there are no significant differences of muscle thickness between rest and MVC which supports the simple planar muscle model [28] and previous results from Manal et al. [29]. In this planar muscle model, it is assumed that muscle thickness is constant in contraction condition. Therefore, our results demonstrate that ultrasound imaging technique is feasible to evaluate the muscle architectural changes after subacute stroke, which could facilitate the understanding of muscle functional recovery after intervention.

There are many studies that had demonstrated the muscle morphology changes after training both in athletes and subjects with neurological insults, which are similar to the findings of this current study. Blazevich et al. found the muscle thickness of rector femoris enlarged from 2.08–2.4 cm to 2.5–2.58 cm and fascicle length increased from 10.6–16 cm to 14.7–21.6 cm in athletes after 5 weeks resistance training [30]. Brorsson and coworkers found the cross-section area of extensor digitorum communis increased after a six-week hand exercise programme in patients with rheumatoid arthritis [31]. In line with these studies, increased muscle thickness in TA and longer fascicle length in MG were also found in subacute stroke after 3-week treadmill training, which may demonstrate that the training could counteract the muscle atrophy and enlarge the muscle volume. In the current study, the muscle strength of dorsiflexion at the affected side at BWSTT group was significantly increased after the training and showed better performance in walking ability (Table 4). Furthermore, the strength of ankle dorsiflexors and ankle plantarflexors obtained in our study (Table 3) is close to Dorsch et al.'s results of stroke survivors (ankle dorsiflexors: 66 ± 37; ankle plantarflexors: 93 ± 53) [32]. This may suggest that with the proper setup, hand-held dynamometry could be applied to measure the muscle strength in patients with neurological diseases, such as stroke. Meanwhile, our results showed that the plantarflexors strength significantly increased in the BWSTT group. This is in line with the study of Brincks and Nielsen who found that instantaneous plantarflexion power and enough work done in the push-off phase ensure the generation of walking speed [33]. In addition, Pradon and coworkers also demonstrated that the muscle strength had significant positive association with walking distance in persons after stroke [34]. These results may indicate that it is clinically meaningful to measure muscle architectural parameters after stroke after exercise training, which help to evaluate the muscle performance and motor function recovery.

**Figure 4:**
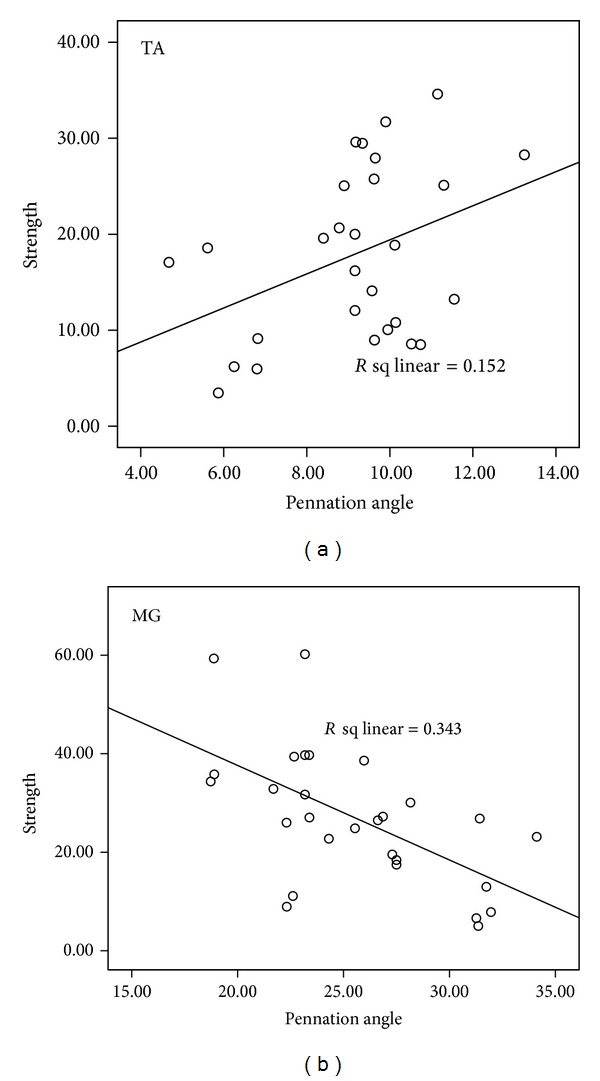
Correlation coefficient results of pennation angle and muscle strength on TA (a) and MG (b) of the affected side of BWSTT group after training.

In order to investigate the relationship between muscle architectural parameters with muscle strength, correlation analysis was performed (Figure 4). The results showed that after the training, there is significant positive correlation between TA pennation angle and muscle strength, and negative correlation between MG pennation angle and muscle strength in the affected side of BWSTT group (Table 5). The relationship between the increased pennation angle and muscle force changes needs further discussion. It is believed that muscle pennation angle has advantageous effects (more muscle fibre and contractile material attached to tendon) [35] and disadvantageous (less efficient force transmission from muscle fibers to tendon) [23] on force generation. Therefore, according to our results, BWSTT facilitates more muscle fibre packed in the same cross-section area which may have larger effects than the force transfer effects to the tendon. That is the reason why the correlation showed a positive relationship in TA. Similarly, Kawakami and coworkers found that load training of upper limb could increase both the pennation angle of triceps brachii and the performance of the subjects [36]. The relation between pennation angle and force might depend on the pennation value itself. If the pennation angle is larger than 20°, it has great significance on the transferred force [21]. This could explain the negative relationship between pennation angle of MG and its muscle strength. Further investigations with other lower limb muscles, that is, hamstring and rector femoris, are warranted to see the effects of pinnate muscle architecture on the muscle force generation ability after stroke.

There are still discussions about training intensities and time window of applying body weight support training to optimize the effects on the outcomes to the stroke survivors. In this study, we applied BWSTT in a group of subacute stroke survivors with average suffering days of 37.5, which is in line with the concept that rehabilitation should be as early as possible to be involved in [37]. Our results demonstrated that after 3 weeks of BWSTT, the patients improved in FMA-LE, MAS, and walking speed (Table 4). Our training effects on walking speed are similar with literature [9]. For example, Franceschini used treadmill training with body weight support on early stage of stroke, and, after 10 sessions of training, their data of 10-meter walking speed is 0.4 m/s (with range of 0.3~0.6 m/s). There is study showing that if too much body weight is supported, the affected side could not get enough stimulus from exercise training and will not benefit the motor recovery of the lower limb function [4]. In the current study, the subject would have an initial body weight support less than 40% which could ensure that the two sides of the lower limb touch on the treadmill and the subjects are safe. During the training process, the weight support will be gradually reduced and all our subjects need not the support in the end of the training. Previous study had showed that the optimal speed should be similar to normal walking speed [38]. In our study, the patients at least had a speed of 1.3 mph in the end of the training which is similar to this finding. Although there is no huge change of vital parameters and observation in our study which may prove that the training is safe for subacute stroke survivors, we will suggest measuring blood pressure and heart rate after each training session.

There are still limitations that need to be discussed in this study about using ultrasound to evaluate muscle architecture. In literature, no consensus has been achieved on the body posture during measurement. We followed muscle testing manual as well as the literature on the body posture and fixation of the hand-held dynamometer on the lower limb. Further study is warranted to evaluate the effects of body posture and limb position on the muscle measurement. Secondly, although the correlation of muscle strength with muscle pennation angles of TA and MG before and after training has significant changes in the results, the correlation coefficient is still relatively small. This might be related to the variation of our patients' improvement on motor recovery after intervention. Further studies with larger sample size or multicenter design are needed to assess the clinical effects of BWST on muscle function in early treatment after stroke. For the future applications of current study, since the individual muscle force could not be measured with a noninvasive way, the relationship between muscle architectural changes as measured in this study and the generated muscle force could not be interpreted directly. Neuromusculoskeletal model has been applied to calculate individual muscle force based on musculotendon parameters and to predict joint movement [39]. Forward dynamic modeling method will be applied to calculate individual muscle force and the results could be used to compare with joint moment measurement for further evaluating the muscle function for persons after stroke.

## 6. Conclusions

This study showed that ultrasound measurement is a feasibility method to evaluate the muscle architectural changes in subacute stage of stroke and there are considerable changes in tibialis anterior and medial gastrocnemius fascicle architecture, which may contribute directly to the impaired lower limb motor functions. BWSTT can improve muscle strength, walking speed, and motor functions of persons with subacute stroke and the functional changes of lower limb are related to muscle architecture of TA and MG. Our results demonstrate that BWSTT is a feasible and effective gait training method for subacute stroke in an early stage.

## Figures and Tables

**Table 1:** Baseline demographic and clinical characteristics of the patients.

Characteristics	BWSTT (*n* = 8)	Control (*n* = 7)	*P* value*
Age (years)	61.63 (8.43)	59.29 (9.11)	0.821
Female	3 (37.5%)	3 (42.86%)	1.00
Height (cm)	165.88 (6.81)	165.71 (7.54)	0.612
Bodyweight (Kg)	60.75 (4.43)	61.14 (5.46)	0.472
Ischemic stroke	2 (25.0%)	2 (28.57)	1.00
Affected side at left	4 (50.0%)	5 (71.43%)	0.608
Days after stroke	45.25 (17.60)	58.71 (19.52)	0.644
FMA-LE	23.13 (4.29)	22.0 (4.51)	0.375
MAS	1.69 (0.26)	1.64 (0.24)	0.738

Data are presented as mean (SD) or *n* (%); BWSTT: body weight support treadmill training; FMA-LE: Fugl-Meyer assessment of lower limb; MAS: Modified Ashworth Scale.

*Based on the independent *t*-test or Fisher's exact test.

**Table:** (a)

TA mean (SD)			BWSTT group		CGT group	
			Affected	Unaffected	Affected	Unaffected
Pennation angle (°)	Rest	Before	6.15 (1.28)	7.60 (2.58)	6.47 (1.26)	6.78 (2.08)
		After	7.26 (1.62)*	7.81 (2.07)	6.05 (0.86)	6.34 (1.55)
	MVC	Before	7.65 (2.20)	9.78 (2.82)	8.16 (1.49)	8.67 (1.57)
		After	9.11 (1.95)*	10.29 (2.46)	7.94 (1.18)	8.55 (1.75)

Muscle thickness (cm)	Rest	Before	1.02 (0.09)	1.14 (0.16)	0.90 (0.16)	1.05 (0.85)
		After	1.09 (1.12)*	1.09 (0.12)	0.86 (0.11)	0.85 (0.10)
	MVC	Before	1.16 (0.07)	1.26 (0.17)	1.02 (0.11)	1.15 (0.16)
		After	1.21 (0.11)*	1.22 (0.14)	1.01 (0.10)	0.93 (0.10)

Fascicle length (cm)	Rest	Before	7.52 (1.90)	7.45 (2.28)	6.48 (0.87)	6.56 (1.43)
		After	7.43 (1.88)	7.28 (1.79)	6.38 (1.01)	6.68 (1.44)
	MVC	Before	7.45 (1.80)	7.18 (2.27)	6.12 (1.25)	5.62 (1.11)
		After	7.13 (2.32)	6.79 (2.04)	6.03 (1.17)	5.47 (0.84)

**Table:** (b)

MG mean (SD)			BWSTT group		CGT group	
			Affected	Unaffected	Affected	Unaffected
Pennation angle (°)	Rest	Before	18.73 (3.44)	19.74 (5.66)	20.82 (6.44)	19.35 (4.21)
		After	19.32 (3.86)	21.35 (4.50)	19.05 (6.70)	22.27 (11.81)
	MVC	Before	26.75 (4.17)	26.12 (5.65)	27.48 (5.29)	26.16 (7.09)
		After	25.57 (4.26)	30.45 (6.89)	28.69 (7.31)	26.68 (7.65)

Muscle thickness (cm)	Rest	Before	1.50 (0.21)	1.60 (0.32)	1.53 (0.31)	1.65 (0.37)
		After	1.61 (0.26)	1.64 (0.32)	1.58 (0.36)	1.65 (0.25)
	MVC	Before	1.59 (0.27)	1.67 (0.27)	1.66 (0.17)	1.69 (0.24)
		After	1.63 (0.27)	1.66 (0.23)	1.65 (0.24)	1.67 (0.13)

Fascicle length (cm)	Rest	Before	4.66 (1.06)	5.13 (1.17)	4.65 (0.66)	5.18 (1.12)
		After	5.23 (1.07)*	5.15 (1.06)	4.84 (0.93)	5.10 (1.21)
	MVC	Before	3.76 (1.08)	3.74 (1.18)	3.74 (0.57)	3.78 (1.0)
		After	4.02 (4.14)*	3.41 (1.04)	3.54 (0.90)	3.44 (0.73)

SD: standard deviation.

**Table:** (a)

Dorsiflexion (*N*) Mean (SD)	Before	After	*t*	*P*
BWSTT affected	49.04 (28.12)	83.75 (42.72)	−4.780	0.000*
BWSTT unaffected	142.85 (32.57)	134.57 (41.70)	0.985	0.333
CGT affected	47.08 (25.01)	55.71 (25.50)	−1.928	0.069
CGT unaffected	129.45 (33.15)	122.78 (40.85)	0.687	0.503

*P < 0.05.

**Table:** (b)

Plantarflexion (*N*) Mean (SD)	Before	After	t	P
BWSTT affected	93.67 (40.94)	115.39 (65.37)	−2.144	0.041*
BWSTT unaffected	188.95 (56.07)	179.78 (75.38)	0.470	0.643
CGT affected	85.48 (43.83)	96.25 (43.83)	−1.348	0.193
CGT unaffected	175.20 (37.87)	173.11 (56.16)	0.202	0.842

*P < 0.05.

SD: standard deviation.

**Table 4:** Comparison of FMA and MAS scores and 10-metre walking speeds before and after training.

		Before	After	*P*
FMA Mean (SD)	BWSTT	23.14 (4.63)	25.57 (4.69)	0.002*
	CGT	21.8 (4.49)	24.2 (2.95)	0.051

MAS Mean (SD)	BWSTT	1.64 (0.24)	1.48 (0.59)	0.038*
	CGT	1.21 (0.24)	1.14 (0.59)	0.095

10-metre walking speeds (m/s)	BWSTT	0.36 (0.15)	0.55 (0.20)	0.004*
	CGT	0.46 (0.21)	0.47 (0.23)	0.854

*P < 0.05.

SD: standard deviation.

**Table 5:** Correlation of muscle strength with muscle pennation angles of TA and MG before and after training.

			Correlation coefficient (*r*)	*P* value
TA	BWSTT	Before	−0.148	0.316
		After	0.390	0.04*
	CGT	Before	−0.291	0.213
		After	0.646	0.002*

MG	BWSTT	Before	−0.259	0.075
		After	−0.586	0.001*
	CGT	Before	−0.141	0.552
		After	−0.312	0.180

*P < 0.05.
